# Estimating the immunogenicity of measles-rubella vaccination administered during a mass campaign in Lao People’s Democratic Republic using multi-valent seroprevalence data

**DOI:** 10.1038/s41598-019-49018-y

**Published:** 2019-08-29

**Authors:** Emilia Vynnycky, Shinsuke Miyano, Katsuhiro Komase, Yoshio Mori, Makoto Takeda, Tomomi Kitamura, Anonh Xeuatvongsa, Masahiko Hachiya

**Affiliations:** 1 Modelling and Economics Unit, Public Health England, 61 Colindale Avenue, Colindale, NW9 5HT London UK; 2 TB Modelling Group and TB Centre, London School of Hygiene & Tropical Medicine, London, UK; 3 Centre for Mathematical Modelling of Infectious Diseases, Faculty of Epidemiology and Population Health, London School of Hygiene & Tropical Medicine, London, UK; 4 Bureau of International Health Cooperation, National Center for Global Health and Medicine, 1-21-1 Toyama, Shinjuku, Tokyo 162-8655 Japan; 5 Department of Virology III, National Institute of Infectious Diseases, 4-7-1 Gakuen, Musaashi-Murayama, Tokyo, 208-0011 Japan; 6 Ministry of Health, National Immunization Program, Simeuang Road, Vientiane, Lao PDR

**Keywords:** Viral infection, Epidemiology

## Abstract

Measles and rubella are important causes of morbidity and mortality globally. Despite high coverage reported for measles vaccination, outbreaks continue to occur in some countries. The reasons for these outbreaks are poorly understood. We apply Bayesian methods to multi-valent seroprevalence data for measles and rubella, collected 2 years and 3 months after a mass measles-rubella vaccination campaign in Lao PDR to estimate the immunogenicity and vaccination coverage. When the vaccination coverage was constrained to exceed 95% or 90%, consistent with officially-reported values, the immunogenicity of the measles vaccine component was unexpectedly low (75% (95% CR: 63–82%) and 79% (CR: 70–87%) respectively. The estimated immunogenicity increased after relaxing constraints on the vaccination coverage, with best-fitting values of 83% (95% CR: 73–91%) and 97% (95% CR: 90–100%) for the measles and rubella components respectively, with an estimated coverage of 83% (95% CR: 80–88%). The findings suggest that, if the vaccine coverage was as high as that reported, continuing measles outbreaks in Lao PDR, and potentially elsewhere, may be attributable to suboptimal immunogenicity attained in mass campaigns. Vaccine management in countries with high reported levels of coverage and ongoing measles outbreaks needs to be reviewed if measles elimination targets are to be achieved.

## Introduction

Measles and rubella are important causes of morbidity and mortality globally. In 2017, approximately 11000 people died from measles^1^, whilst 105,000 cases of Congenital Rubella Syndrome - associated with lifelong disability (including deafness, cataracts, cardiac defects and mental retardation) in a child following infection acquired by its mother during pregnancy – occurred in 2010^2^. Despite levels of coverage reported for measles vaccination of above the herd immunity threshold (the coverage required to control transmission^3^), measles outbreaks continue to occur in some countries. In Lao PDR, for example, supplemental immunization campaigns (SIAs) of measles and the measles-rubella vaccine reported coverage of 96% in 2007 and 97% in 2011 respectively, targeting wide age ranges (children aged 9 months-14 years and 9 months – 19 years respectively), and outbreaks were reported in 2012, 2013 and 2014^4^. The factors contributing to these ongoing outbreaks are poorly understood, but need to be elucidated if targets specified in the Global Vaccine Action Plan of measles elimination in 5 world regions by 2020^5^ are to be achieved.

One potential factor contributing to these outbreaks is a low immunogenicity of the measles vaccine, which could lead to suboptimal levels of immunity in populations with high reported levels of coverage. One recent study of measles-rubella IgG seroprevalence in Lao PDR two years and three months after a measles-rubella vaccination campaign found surprisingly different levels of seroprevalence for measles and rubella and suggested that the heat stability of the measles and rubella components of the vaccine differed^4^. Insufficient immunogenicity of the measles component of the vaccine could result from suboptimal vaccine handling. There are no direct estimates of the immunogenicity of the measles component of vaccines administered in populations during campaigns.

In the absence of direct measurements, insight into the immunogenicity of different components of a vaccine may be obtained by estimating the proportion of previously seronegative people who seroconvert, using statistical analyses of multi-variate sera among people who have received a multi-valent vaccine. Such analyses also allow estimation of the vaccination coverage, allowing verification of reported levels of coverage. To our knowledge, the only studies to date which have estimated the immunogenicity using such analyses considered the measles-mumps-rubella (MMR) vaccine in Western countries and found levels of seroconversion of at least 94% for the measles and rubella components^6,7^, even when adjusting for possible waning in seroprevalence following vaccination^7^.

In this paper we estimate the proportion of people who seroconverted to the measles and rubella components of the measles-rubella vaccine administered during the campaign in 2011 in Lao PDR using data on the proportion of people who were seropositive for measles and/ or rubella in a survey conducted in 2014.

## Results

Table 1 summarizes estimates of the percentage of seronegative people who, after vaccination, became positive for measles and rubella antibodies and the estimated average vaccination coverage. For the base case assumptions (>95% vaccination coverage) and when estimated just using data for 5–14 year olds, this percentage was lower for measles (75%, 95% CR: 63–82%) than for rubella (89%, 95% CR: 80–96%).

**Table 1:** Summary of the estimated percentage of seronegative people who became positive for measles or rubella antibodies after vaccination and the vaccine coverage, based on 1000 million initial samples and 50,000 resamples.

Age group considered in the estimation	Constraint on the vaccine coverage	Estimated % of seronegative people who become positive after vaccination for antibodies to:		Estimated average vaccination coverage (%)	Deviance
		Measles (*e*_*m*_)	Rubella (*e*_*r*_)		
5–14 year olds	>95 (basecase)	74.6 (62.7,81.9)	89.4 (79.7,95.6)	95.3 (95,96.5)	61
	>90	78.9 (69.5,86.5)	94.8 (87.8,98.4)	90.5 (90,92.4)	46
	>80	82.9 (72.9,91.5)	96.7 (89.7,99.7)	82.7 (80.1,87.8)	37
	>50	85.1 (73.1,97.7)	96.6 (89.8,99.7)	79.3 (67.4,86.6)	38
	>0	86.5 (74.4,98.2)	96.4 (89.7,99.9)	79 (66.9,86.8)	38
15–21 year olds	>95 (basecase)	88.1 (77.2,93.7)	75.5 (56.9,85.2)	96.4 (95.1,99.3)	26
	>90	89.6 (78.4,96)	75.9 (57.1,86.4)	93.7 (90.2,98.5)	26
	>80	90.4 (77.8,98.3)	75.9 (56,87.9)	91.8 (82.1,98.1)	26
	>50	90.4 (77.3,98.5)	76.1 (56.5,89.1)	91.7 (78,98.1)	26
	>0	90.4 (77.3,98.6)	75.9 (56.2,88.8)	91.7 (78,98)	26
All age groups	>95 (basecase)	75.6 (72.3,81.3)	88.4 (77.3,88.4)	95.6 (95.1,96.1)	115
	>90	83.1 (71.6,87)	87.5 (83.3,93.1)	90.4 (90.1,92.8)	103
	>80	82.1 (79,90.8)	91.4 (88.5,95)	85.5 (81.1,88.5)	87
	>50	86.1 (86.1,89.6)	94.2 (94.2,94.4)	81.6 (76.9,81.6)	85
	>0	86 (76.2,90.9)	91.6 (83.5,99.6)	81.7 (74.9,89.7)	96

The values in parentheses show the 95% credible range. The number of degrees of freedom when considering data for 5–14, 15–21 and 5–21 year olds was 7, 4 and 14 respectively. The deviance reflects the minimum deviance seen in the 50,000 resamples.

**Figure 1:**
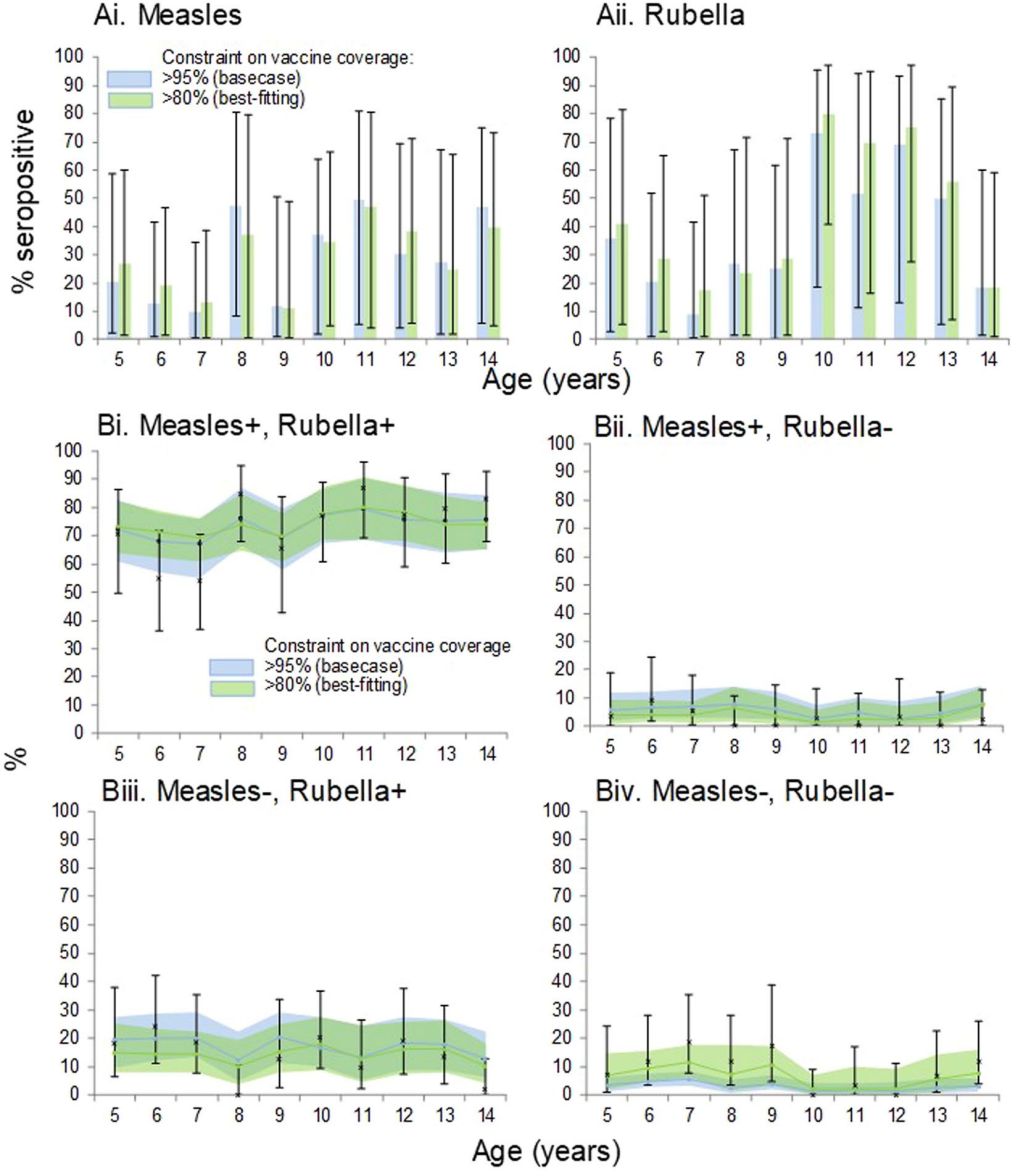
Comparison between base-case estimates (>95% vaccination coverage) and best-fitting estimates (>80% vaccination coverage). (**A**) Age-specific proportion of unvaccinated people who had acquired detectable antibodies to measles (*c*_*a,m*_) or rubella (*c*_*a,r*_) through natural infection (measles and rubella) or vaccination (measles only). The bars show the 95% credible range. (**B**) Comparison between the observed data (crosses, with bars for the 95% CI) and the % estimated to be positive or negative for measles and/or rubella antibodies. The solid squares and shaded areas respectively show the median and 95% credible range of the calculated estimates.

As the lower limit on the prior for the estimated vaccination coverage decreased to 80%, the estimated percentage of seronegative people who, after vaccination, became seropositive increased, reaching 83% (95% CR: 73–91%) and 97% (95% CR: 90–100%) for measles and rubella antibodies respectively. High values (>85%) for the immunogenicity, with upper limits for the credible range exceeding 95%, were estimated when the vaccination coverage was allowed to be low in the estimation (Table 1). For example, when the vaccination coverage was allowed to range between 0% and 100% in the estimation, the estimated immunogenicity was 86% (95% CR: 74–98%) and 96% (95% CR: 90–100%) for the measles and rubella components respectively, with an average vaccination coverage of 79% (95% CR: 67–87%) (Table 1). The best-fit was obtained assuming that the coverage was over 80% (deviance = 37), which was similar to that obtained either without constraining the coverage or constraining it to be >50% (deviance of 38), whilst the worst fit resulted from assuming that the coverage was over 95% (deviance = 61) (Table 1 and Fig. 1). Both the best-fitting and base-case estimates led to a wide range for the percentage of people who were seropositive for measles or rubella antibodies because of natural infection or vaccination before the SIA (Fig. 1A).

When applied to data for all age groups simultaneously, for a given constraint on the vaccine coverage, the Bayesian Melding approach resulted in similar estimates for the vaccine coverage and immunogenicity of the measles and rubella components as those obtained when just considering data for 5–14 year olds, with the best fit overall obtained by constraining the vaccine coverage to be >80%. When applied just to data for 15–21 year olds, the fit of the model was similar (deviance = 26) and the estimated average vaccine coverage was over 90%, irrespective of the constraint applied on the vaccine coverage.

The same patterns were also seen when the unknown parameters were fitted by maximum likelihood (Table 2 and Fig. A.2 (Supporting Information)). For example, the best-fitting percentage of seronegative people who, after vaccination, became positive for measles antibodies was below 90% when the vaccine coverage was constrained to be above 90 or 95% and the best-fit was obtained when the vaccination coverage was constrained to be either >0, >50 or >80% (deviance = 49). For the latter scenario, 84% (95% CR: 73–94%) and 100% (95% CR: 92–100%) of seronegative people were estimated to become positive for measles and rubella antibodies respectively after vaccination, and the best-fitting vaccine coverage was 81% (95% CR: 80–90%).

**Table 2:** Summary of the estimated percentage of seronegative people who became positive for measles or rubella antibodies after vaccination and the estimated vaccination coverage, obtained by fitting to the observed data using maximum likelihood.

Age group considered in the estimation	Constraint on the vaccine coverage	Estimated % of seronegative people who become positive after vaccination for antibodies to:		Estimated average vaccination coverage (%)	Deviance
		Measles (*e*_*m*_)	Rubella (*e*_*r*_)		
5–14 years	>95% (basecase)	83.6 (76,87.9)	95.8 (92.1,98.4)	95 (95,95)	56
	>90%	83.2 (71.5,89.8)	96.8 (91.4,100)	90 (90,90.7)	50
	>80%	84.3 (72.9,94.2)	99.9 (92,100)	80.6 (80,90.2)	49
	>50%	84.7 (74.1,99.9)	99.1 (92.3,100)	81.3 (63.8,89.8)	49
	>0%	84.7 (73.7,99.9)	99.1 (92.5,100)	81.3 (64,89.4)	49
15–21 years	>95% (basecase)	86.6 (73.1,94.9)	87.4 (66.4,92.1)	95 (95,99.4)	33
	>90%	88.6 (68.1,96.9)	81.2 (65.2,96.2)	90 (90,99.4)	33
	>80%	86.9 (61,98.4)	87.3 (61.5,100)	95.2 (80,99.2)	33
	>50%	84.8 (65.5,99.9)	87.8 (57.9,100)	94 (64.7,99.3)	33
	>0%	76.7 (64,99.9)	96.2 (60.5,100)	75.6 (65.3,99.3)	33
All age groups	>95% (basecase)	76.8 (67.2,85.2)	92.7 (89.9,95.2)	95 (95,95)	109
	>90%	75.1 (68.1,81.6)	97.1 (94.3,99.6)	90 (90,90)	94
	>80%	75.2 (67.8,83.7)	100 (97.5,100)	84.7 (80,88.2)	90
	>50%	75.2 (68.3,99.9)	100 (97.3,100)	84.7 (63,88.2)	90
	>0%	75.2 (68.3,99.8)	100 (97.4,100)	84.7 (63.9,88.2)	90

The values in parentheses show the 95% confidence intervals, based on 1000 bootstraps. The number of degrees of freedom when considering data for 5–14, 15–21 and 5–21 year olds was 23, 14, and 14 respectively.

As shown in Table 3 and Figs A.2–A.4 (Supporting Information), when applied to simulated data for 5–14 year olds and using priors which were consistent those assumed when generating the data, the Bayesian Melding approach led to estimates of the immunogenicity and vaccine coverage which were consistent with those assumed, with the 95% credible range spanning the assumed value. When applied just to data for 15–21 year olds or all age groups, although the parameter estimates were typically consistent with those assumed, they sometimes overestimated them, particularly when the vaccine coverage used to generate the data was high.

**Table 3:** Estimated percentage of seronegative people who became positive for measles or rubella antibodies after vaccination and the vaccine coverage obtained by applying the Bayesian Melding approach (1000 M initial samples and 50,000 resamples) to simulated data, in which the vaccine coverage was assumed to be 77%, 85% or 97%. The values in parentheses show the 95% credible range.

Assumed vaccine coverage in the simulated data	Constraint on the estimated vaccine coverage	Age group considered in the estimation	Estimated % of seronegative people who become positive after vaccination for antibodies to:		Estimated vaccination coverage (%)	Deviance
			Measles (*e*_*m*_)	Rubella (*e*_*r*_)		
77% (low)	>50%	5–14 years	83.4 (70.4,94)	82.9 (68.4,93.1)	88.1 (74.8,95.6)	6
		15–21 years	81.4 (63.6,89.7)	88.8 (75.7,95.5)	97 (87.5,99.8)	3
		All age groups	84.1 (73.3,88.6)	83.6 (76.9,89.6)	93.2 (86.4,96.9)	26
85% (medium)	>80%	5–14 years	84.9 (72.9,92.9)	86.7 (75.3,94.5)	93.3 (84,98.5)	7
		15–21 years	78.5 (61,88.4)	92.5 (81.6,98.9)	93.8 (84,98.8)	3
		All age groups	84.5 (74.4,89)	89.3 (83.9,93.5)	94 (89.5,97.5)	21
97% (high)	>95%	5–14 years	84.1 (72.7,90.2)	90.9 (83,95.3)	99.2 (96.1,100)	6
		15–21 years	92.2 (82.2,96.6)	93.8 (85.5,97.5)	99.2 (95.9,100)	4
		All age groups	87.5 (81.5,91.5)	92.5 (87.8,95.4)	99.6 (97.8,100)	19

The average values for the immunogenicity of the measles and rubella components of the vaccine (*e*_*m*_ and *e*_*r*_), used in simulating the data were 75% and 90% respectively. The number of degrees of freedom when fitting using data for 5–14, 15–21 and 5–21 year olds was 7, 4 and 14 respectively. The deviance reflects the minimum deviance seen in the 50,000 resamples.

## Discussion

Our analysis suggests that one possible explanation for continuing outbreaks of measles following the mass measles-rubella vaccination campaign in 2011 in Lao PDR, if the coverage attained was as high as that reported, is that the immunogenicity was sub-optimal. In the base-case, with an assumed vaccination coverage consistent with that reported (>95%^4,8^), 75% (95% CR: 63–82%) of vaccinees were estimated to become positive for measles antibodies, which is considerably lower than the corresponding levels of over 88% which have been estimated for the MMR vaccine in studies and trial conditions elsewhere^9^. The immunogenicity for the rubella component of the vaccine was consistent with the 93% estimated elsewhere^9^, at least when the estimation was applied just to data for 5–14 year olds.

**Figure 2:**
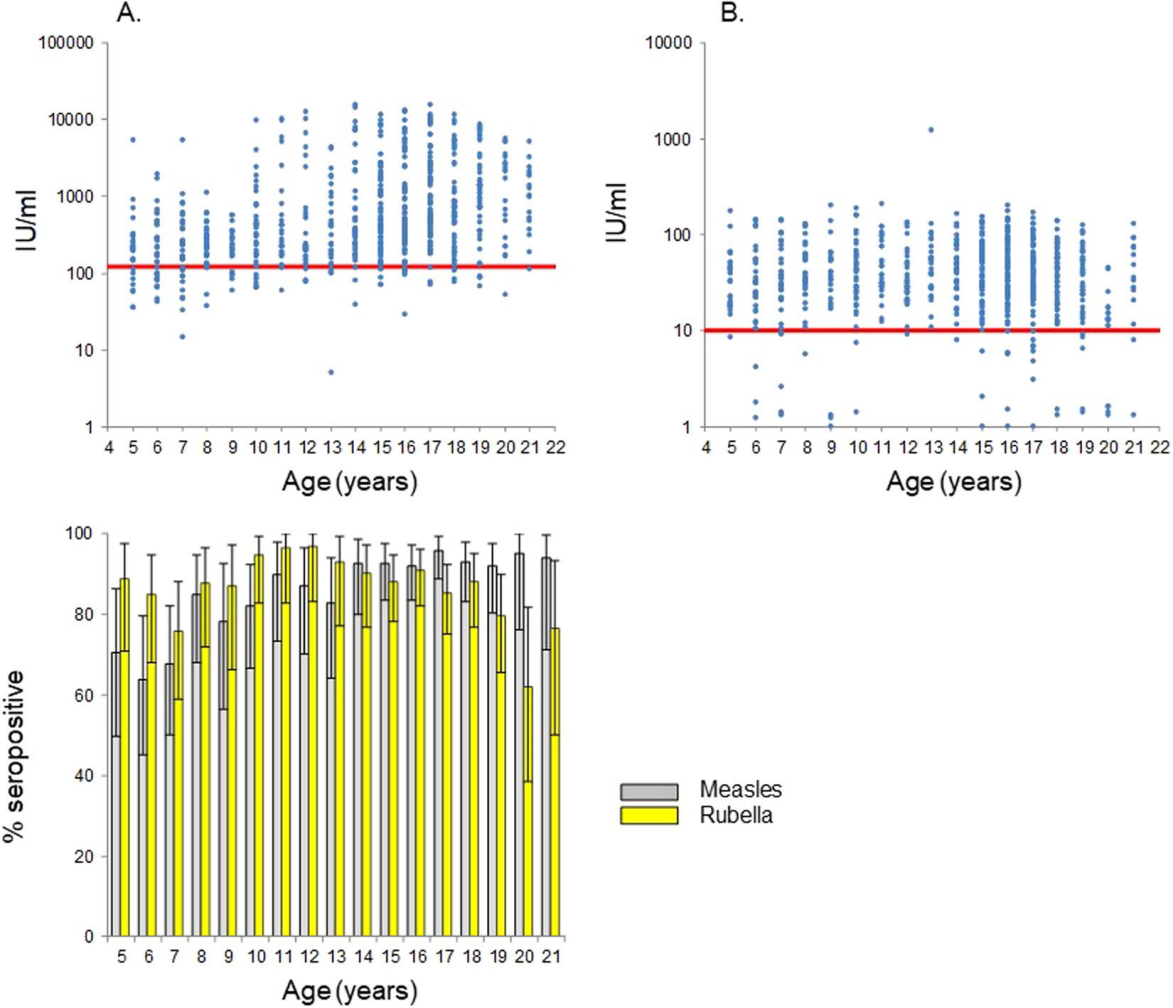
(**A**) Measles and (**B**) Rubella antibody titres for the study population. The red lines show the threshold for defining seropositivity (120 IU/ml and 10 IU/ml for measles and rubella respectively). (**C**) Age-specific percentage seropositive for measles and rubella. The bars show 95% confidence limits on the observed data.

Our analyses relied on several simplifying assumptions. For simplicity, the proportion of people who seroconverted after vaccination was assumed to be the same for all ages, whereas it could potentially vary by age. In the absence of serial seroprevalence data, we did not account for the possibility of immunity waning over time. Recent estimates considering serial data from Australia and observational studies suggest that very few (typically <1%) vaccinees lose their immunity each year^7^. Studies have found that antibody titres decrease with time since vaccination for measles^10,11^, particularly in the absence of natural boosting^11^, and that the proportion who remain seropositive changes little over time. Consequently, our assumption that immunity did not wane over time would have influenced our findings only if the antibody titres induced by vaccination were relatively close to the level required for protection. As shown in Fig. 2, this is unlikely to have been the case since most study participants considered to be seronegative for measles had measles antibody titres more than 10IU/ml lower than the threshold required for protection. Given the slow decline in vaccine-induced antibodies, they are unlikely to have had sufficient levels for protection at time of vaccination.

Our study suggests that the observed data in Laos could have resulted from high levels of vaccine coverage but low levels for the immunogenicity of the measles component of the vaccine, or from low levels of vaccine coverage but high levels for the immunogenicity of the measles component of the vaccine. It is difficult to rule out either of the two possibilities.

For example, previous estimates of the immunogenicity of the measles and rubella vaccine components in multivalent vaccines have typically exceeded 90% but in our analyses, such high levels for the measles component were attainable only when the vaccination coverage was allowed to be below 90%. Such levels of coverage are plausible as they have been reported for routine measles vaccination. In addition, low levels of coverage and high immunogenicity led to a better fit to the observed data than estimates of high levels of coverage and low levels for the immunogenicity, although the interpretation of the fit is complicated by the low number of degrees of freedom associated with the estimates (Table 1).

On the other hand, high levels of coverage are consistent with the levels of coverage reported during the two most recent SIAs in Lao PDR (in 2007 and 2011), namely 96% and 97% respectively^4^. Low levels for the immunogenicity of the vaccine are also consistent with reports^4^ of field work during the survey, which suggested that vaccine management was sometimes suboptimal, with occasional inadequate handling of vaccine vials and incorrect preparation of vaccines (e.g., keeping vials out of the cold chain for several weeks or filling the vaccine into the syringe the day before injection). Another recent study of cold chain management in two regions in Lao PDR found that whilst vaccines were stored at appropriate temperature provincially, they frequently experienced overheating or freezing both during storage at district level and transportation^12^. Elucidation of the true vaccine coverage and immunogenicity would require serological testing shortly before and after administering the vaccine, as has been done in some studies^13^.

Although our estimates of the immunogenicity for the measles component of the vaccine were lower than for the rubella component (75% (95% CR: 63–82%) vs 89% (95% CR: 74–96% in the basecase) respectively, the difference was not statistically significant. Differences in the immunogenicity between the two would be consistent with recent findings of differences in the heat-stability between the measles and rubella components of the measles-rubella vaccine used during the SIA in 2011, under certain conditions. For example, a reduction of almost 85% in the infectious titre of the measles component of the freeze-dried vaccine was seen after being incubated for 28 days at 35 °C, whereas no reduction was seen at reduced temperatures (4 °C and 25 °C). In contrast, no reduction was seen for the corresponding rubella component at any of the temperatures tested (4 °C, 25 °C and 35 °C).

In conclusion, our analysis suggests that one possible explanation for continuing outbreaks of measles following the mass measles-rubella vaccination campaign in 2011 in Lao PDR, if the levels of coverage were as high as those reported, is a low immunogenicity 75% (95% CR: 63–82%) of the measles component of the vaccine. Our findings are likely to be relevant for other countries, which are experiencing measles outbreaks despite high levels of reported coverage. Vaccine management in such countries needs to be reviewed if measles elimination targets are to be achieved.

## Methods

### Data sources

We used data collected during a nationwide multistage random cluster sampling survey in 2014 in Lao PDR, measuring anti-measles and anti-rubella IgG prevalence among children and adults, as described in^4^. The survey collected blood samples from 2135 children and adults living in 52 randomly-selected villages drawn from all 143 districts of Lao PDR and was conducted for 2 weeks from 27 January to 7 February, 2014. The analyses were restricted to data for the age groups 5–21 years as people in this age range would have received MR vaccine in 2011. As described in^4^, IgG levels were measured from dried blood spots on the filter paper using commercially available enzyme-linked immunosorbent assay (ELISA) kits (Enzygnost Anti-Measles Virus/IgG and Anti-Rubella Virus/IgG, Siemens Healthcare Diagnostics) according to the manufacturer’s instructions at the Department of Virology 3, National Institute of Infectious Diseases, Japan. Optical density values were converted to quantitative data, and the results were considered positive at higher than 120 mIU/mL for measles and 10 IU/mL for rubella^14,15^. Figure 2 summarises the observed data.

### Estimating the age-specific vaccination coverage and the seroprevalence before the SIA in 2011

#### Expressions for the age-specific proportion positive or negative for measles and/or rubella antibodies

We use the notation *p*_*a,m,r*_ for the proportion in age group *a* who are positive or negative for measles and/or rubella antibodies in 2014, where *m* is replaced by the symbols + or - when referring to those who are positive or negative respectively for measles antibodies and *r* is replaced by the symbols + or - when referring to those who are positive or negative respectively for rubella antibodies. Following the approach of Gay^16^ (unpublished) (described in Altmann and Altmann^17^) and Goeyvaerts *et al*.^6^, it can be shown (Supporting Material) that *p*_*a,m,r*_ can be expressed in terms of the following factors:

1. The vaccination coverage (*v*_*a*_) in age group *a* during the SIA in 2011, assumed to be identical for all age groups..
2. The proportion of people in age group *a* who were not vaccinated during the SIA in 2011 who acquired antibodies to infection *i* (denoted *c*_*am*_ and *c*_*ar*_ for measles and rubella respectively), assumed to differ between each single year age band. For measles, these people would have become seropositive because of natural infection or vaccination; for rubella, they would have become seropositive because of natural infection.
3. The proportion of people who were negative for antibodies to infection *i* just before the SIA in 2011, but then became positive for antibodies to infection *i* because of vaccination, assumed to be identical for all ages (denoted *e*_*m*_ and *e*_*r*_ for measles and rubella respectively). This proportion is interpretable as the immunogenicity of the corresponding component of the vaccine.

The equations are as follows:

1 $${p}_{a++}={v}_{a}{b}_{a,m}{b}_{a,r}+(1-{v}_{a}){c}_{a,m}{c}_{a,r}$$

2 $${p}_{a--}={v}_{a}(1-{b}_{a,m})(1-{b}_{a,r)}+(1-{v}_{a})(1-{c}_{a,m})(1-{c}_{a,r})$$

3 $${p}_{a+-}={v}_{a}{b}_{a,m}(1-{b}_{a,r})+(1-{v}_{a}){c}_{a,m}(1-{c}_{a,r})$$

4 $${p}_{a-+}={v}_{a}(1-{b}_{a,m}){b}_{a,r}+(1-{v}_{a})(1-{c}_{a,m}){c}_{a,r}$$

where *b*_*a,i*_ is the proportion of people in age group *a* who were vaccinated in 2011 and have detectable antibodies for infection *i* in 2014, given by the following equation:

$${b}_{a,i}={e}_{i}+(1-{e}_{i}){c}_{a,i}$$

#### Estimation approach

The unknown parameters were estimated considering the age groups 5–14, 15–21 and 5–21 years separately, Considering all single year age groups in the age range 5–14 years, *p*_*a,m,r*_ can be expressed in terms of 23 unknown parameters (*v*, *c*_*am*_ and *c*_*ar*_ for each single year age band, and *e*_*m*_ and *e*_*r*_), with 17 and 37 unknown parameters when the age groups 15–21 and 5–21 years are considered separately. The large number of unknown parameters complicates the estimation procedure for widely-used approaches, including maximum likelihood and MCMC. We therefore obtained estimates and credible intervals of the unknown parameters by an importance sampling approach often referred to as Bayesian Melding, which is often applied to models involving large numbers of parameters^18–21^. Following this approach, we randomly sampled a sufficiently large number (1000 million) combinations of the parameters, subject to various constraints (see below) and computed the likelihood of the observed age-specific proportion of people who were seropositive and/or negative to measles and/or rubella antibodies. We then resampled 50,000 parameter combinations using the likelihood as the weight. The estimate of the parameter was taken as the median in the resampled combinations and the 95% credible range (CR) was taken as the 95% range of the resamples. To verify that the number of samples used to calculate the estimates was sufficiently large, we compared the estimates obtained against those obtained after repeating the calculations using fewer than 1000 million initial samples and more than 50000 resamples.

In the base case, the sampling was carried out assuming that the vaccination coverage exceeded 95%, consistent with official reports of 97% coverage during the SIA, also constraining on the proportion of those seronegative for antibodies to a given antigen just before the SIA who seroconverted after vaccination to be greater than 50%. The fit of the models resulting from different assumptions was compared using the minimum of the loglikelihood deviance from the 50,000 resamples. In sensitivity analyses, we explored the effect of relaxing the constraints on the vaccination coverage, allowing it to be unconstrained or above 50%, 80% or 90%. No constraints were imposed on the proportion of people who were not vaccinated in 2011, but who were seropositive for antibodies to a given antigen in 2011.

#### Validating estimates of the unknown parameters

To validate the method for estimating the unknown parameters, we also estimated them using maximum likelihood, by minimising the loglikelihood deviance, after reducing the number of unknown parameters to 7 by constraining the age-specific proportion of people who were not vaccinated during the SIA in 2011 who acquired antibodies to infection *i* (*c*_*am*_ and *c*_*ar*_ for measles and rubella respectively) to change linearly with age. The fitting was implemented using an algorithm based on the simplex method of Nelder and Mead, written in the C programming language^22^. 95% confidence intervals on the estimated parameters were calculated using non-parametric bootstrap, based on 1000 bootstrap datasets, adapting the approach used Shkedy *et al*.^23^ to use multinomial data (Supporting Material).

In addition, we applied the Bayesian melding approach to three simulated datasets in which the average vaccination coverage was either 77%, 85% or 97%, the remaining parameters are the same in each dataset, and the immunogenicity of the measles and rubella components of the vaccine were 77% and 90% respectively. Table A.1 (Supporting Information) summarizes the simulated datasets. As was the case for the observed data, the estimation was carried out for the age groups 5–14, 15–21 and 5–21 years separately.

### Software

The methods were implemented using a specially-written C program, using published routines,.(e.g. the ran1 and indxx functions for random sampling and sorting)^22^.

### Ethical considerations

Ethical considerations for the collection of the data used in this study are described in^4^ and briefly summarised here. All methods were carried out in accordance with relevant guidelines and regulations. To ensure compliance with survey procedures, team members were trained and supervised by national government staff from the National Immunization Programme, National Centre for Laboratory and Epidemiology, the Ministry of Health and provincial health officers, Lao PDR. All experimental protocols were approved by the National Center for Global Health and Medicine (Japan, NCGM-G-001459–00), the ethics committee of the Ministry of Health (Lao PDR, 025-NECHR), and the National Institute of Infectious Diseases (Japan, NIID-494). Written informed consent was obtained from all selected participants. When participants were aged <15 years, they were explained the survey objectives and procedures according to their level of understanding, and informed consent was obtained from their parents or legal guardians.

## Supplementary information

Supplementary material

## Data Availability

Requests for the data should be made to the senior author, Dr Masahiko Hachiya.
