# Supplementary material for: Estimating the immunogenicity of measles-rubella vaccination administered during a mass campaign in Lao People’s Democratic Republic using multi-valent seroprevalence data
Source: Sci Rep. 2019 Aug 29;9:12545. doi: 10.1038/s41598-019-49018-y (PMC6715652; doi:10.1038/s41598-019-49018-y)
Supplement: Supplementary file 1 — Supplementary material [file 41598_2019_49018_MOESM1_ESM.docx]

**Estimating the immunogenicity of measles-rubella vaccination administered during a mass campaign in Lao People’s Democratic Republic using multi-valent seroprevalence data**

**Supplementary Material**

Emilia Vynnycky^1,2,3*^, Shinsuke Miyano^4^, Katsuhiro Komase^5^, Yoshio Mori^5^, Makoto Takeda^5^, Tomomi Kitamura^4^, Anonh Xeuatvongsa^6^, Masahiko Hachiya^4^

* Correspondence to [Emilia.vynnycky@phe.gov.uk](mailto:Emilia.vynnycky@phe.gov.uk)

**Affiliations:**

1. Modelling and Economics Unit, Public Health England, 61 Colindale Avenue, Colindale, NW9 5HT, London, UK

2. TB Modelling Group and TB Centre, London School of Hygiene & Tropical Medicine, London, UK.

3. Centre for Mathematical Modelling of Infectious Diseases, Faculty of Epidemiology and Population Health, London School of Hygiene & Tropical Medicine, London, UK.

4. Bureau of International Health Cooperation, National Center for Global Health and Medicine, 1-21-1 Toyama, Shinjuku, Tokyo 162-8655, Japan

5. Department of Virology III, National Institute of Infectious Diseases, 4-7-1 Gakuen, Musaashi-Murayama, Tokyo 208-0011, Japan

6. Ministry of Health, National Immunization Program, Simeuang Road, Vientiane, Lao PDR.

**Expressions for the proportion of people who are negative or positive for measles and/or rubella antibodies**

We adapt the reasoning used by Gay ^1^, described in Altmann and Altmann^2^, and Goeyvaerts et al^3^, which considered the proportion of people who would be positive for measles, mumps and rubella antibodies antibodies and for consistency, use the notation of Gay ^1^ for the symbols where possible.

Expression for *p_a++_* and *p_a--_*

The proportion of people in age group *a* who are positive for both measles and rubella antibodies in 2014 (*p_a++_*) is given by the sum of two terms, as follows:

*p_a++_* =

Proportion of people in age group a vaccinated in 2011 who have detectable antibodies to measles and rubella

+

Proportion of people not vaccinated in 2011 who have detectable antibodies to measles and rubella because of natural infection or previous vaccination.

The first term in this expression (the proportion of people in age group *a* vaccinated in 2011 who have detectable antibodies to measles and rubella), in turn, is given by the product of the vaccination coverage in age group a and the proportion of those vaccinees who have detectable antibody to the given infection (denoted by *b_a,m_* and *b_a,r_* respectively), i.e. *v_a_ × b_a,m_ × b_a,r_*. We describe the derivation of *b_a,m_* and *b_a,r_* below.

The second term in this expression (the proportion of people in age group *a* not vaccinated in 2011 who have detectable antibodies because of natural infection (measles or rubella) or previous vaccination (measles) in turn is given by the product of three terms, namely the proportion of people in age group *a* who escaped vaccination (1- *v_a_*), the proportion of people aged *a* not vaccinated in 2011 who had acquired detectable antibodies to measles because of natural infection or previous vaccination (*c_a,m_*), and the proportion of people aged *a* not vaccinated in 2011 who had acquired detectable antibodies to rubella because of natural infection (*c_a,r_*), i.e. . (1-*v_a_* )*× c_a,m_ × c_a,r_*.

Therefore, the proportion of people in age group *a* who are positive for both measles and rubella antibodies in 2014 is given by the following equation:

$$p_{a++}=v_{a}b_{a,m}b_{a,r}+(1-v_{a})c_{a,m}c_{a,r}$$

By analogy, proportion of people in age group *a* who are negative for both measles and rubella antibodies in 2014 is given by the following equation:

$$p_{a--}=v_{a}(1-b_{a,m})(1-b_{a,r)}+(1-v_{a})(1-c_{a,m})(1-c_{a,r})$$

Expression for *p_a+-_* and *p_a-+_*

By a similar argument to that used above, the proportion of people in age group *a* who are positive for measles antibodies but negative for rubella antibodies in 2014 is given by the sum of two terms, as follows:

*p_a+-_ =*

Proportion of people vaccinated in 2011 who have detectable antibodies to measles but not to rubella

+

Proportion of people not vaccinated in 2011 who have detectable antibodies to measles but not to rubella

By applying a similar argument to that used for *p_a++_* , the first term in this expression is given by the product of the vaccination coverage in age group *a* and the proportion of those vaccinated in 2011 who have detectable antibody to measles (*b_a,m_*) and the proportion of those vaccinated in 2011 who do not have detectable antibody to rubella (1-*b_a,r_*), i.e. *v_a_ × b_a,m_ × (1-b_a,r_*).

Similarly, the second term in this expression is given by the product of the proportion of people in age group *a* who escape vaccination (1- *v_a_*), the proportion of people aged *a* not vaccinated in 2011 who have acquired detectable antibodies to measles because of natural infection or previous vaccination (*c_a,m_*) , and the proportion of people aged *a* not vaccinated in 2011 who had not acquired detectable antibodies to rubella because of natural infection (1-*c_a,r_*), i.e. *v_a_ × c_a,m_ ×* (1-*c_a,r_*  ).

Therefore, the proportion of people in age group *a* who are positive for measles antibodies but negative for rubella antibodies in 2014 is given by the following equation:

$$p_{a+-}=v_{a}b_{a,m}(1-b_{a,r)}+(1-v_{a})c_{a,m}(1-c_{a,r})$$

By analogy, the proportion of people in age group *a* who are negative for measles antibodies but positive for rubella antibodies in 2014 is given by the following equation:

$$p_{a-+}=v_{a}\left( 1-b_{a,m} \right)b_{a,r}+\left( 1-v_{a} \right)\left( 1-c_{a,m} \right)c_{a,r}$$

Expressions for *b_a,m_* and *b_a,r_*

The proportion of people of age *a*, who were vaccinated in 2011 who have detectable antibodies to measles in 2014 (*b_a,m_*) is given by the sum of two terms:

Proportion of people, previously with no detectable antibody to measles, who acquired detectable antibodies to measles when vaccinated in 2011 (*e_m_*)

+

Proportion of people, previously with no detectable antibody to measles, who **did not** acquire detectable antibodies to measles when vaccinated in 2011 (1-*e_m_*)

×

Proportion of people not vaccinated in 2011 of age *a* who have acquired detectable antibodies because of natural infection or previous vaccination (*c_a,m_)*

*b_a,m_* is therefore given by the following equation:

$$b_{a,m}=e_{m}+(1-e_{m})c_{a,m}$$

By a similar argument to that used for *b_a,m_*, the proportion of people aged *a* who were vaccinated in 2011 who have detectable antibodies to rubella in 2014 (*b_a,r_*) is given by the following equation:

$$b_{a,r}=e_{r}+(1-e_{r})c_{a,r}$$

**Expression for the likelihood of observing the dataset**

We use the following multinomial likelihood of observing the numbers of people who were positive and/or negative for measles and/or rubella antibodies in the dataset:

$$L=\prod_{a} \frac{N_{a}!}{n_{a++}!n_{a+-}!n_{a-+}!n_{a--}!}p_{a++}^{n_{a++}}p_{a+-}^{n_{a+-}}p_{a-+}^{n_{a-+}}p_{a--}^{n_{a--}}$$

Where

*N_a_* is the number of people in age group a who were tested;

*n_amr_* is the observed number of people in age group a who are positive or negative for measles and/or rubella antibodies in 2014, where *m* is replaced by + or - when referring to those who are positive or negative respectively for measles antibodies and *r* is replaced by + or - when referring to those who are positive or negative respectively for rubella antibodies.

### Calculating 95% CI on the parameters estimated by maximum likelihood

95% confidence intervals (CI) for the unknown parameters were calculated using non-parametric bootstrap, based on 1000 bootstrap datasets, adapting the approach used Shkedy et al^4^ to use multinomial data

With this approach, a single bootstrap dataset *B* (*B*=1..1000) comprised *N_ja_* people in age group a, as in the observed data. with $K_{amr}^{B}$ people in age group a who are positive or negative for measles and/or rubella antibodies in 2014, where *m* is replaced by + or - when referring to those who are positive or negative respectively for measles antibodies and *r* is replaced by + or - when referring to those who are positive or negative respectively for rubella antibodies.

$K_{amr}^{B}$ was obtained by first assigning the status “positive for both measles and rubella antibodies”, “positive for measles but negative for rubella antibodies”, “negative for measles but positive for rubella antibodies” and “negative for both measles and rubella antibodies” to $n_{a++}$, $n_{a+ -}$, $n_{a-+}$ and $n_{a--}$ people respectively to *N_a_* people in age group *a* in the dataset, and then drawing *N_a_* samples with replacement from this population. $K_{amr}^{B}$ then equalled the number of people in the *N_a_* samples who had status m, r.

The fitting was repeated for each of the bootstrap datasets and the 95% CI for the unknown oarameters were calculated as their 95% range obtained from the *B*=1..1000 bootstrap datasets.

Table A.1: Summary of the simulated data used in the analyses. Columns 2-6 show the assumptions for the age-specific proportion of people who were not vaccinated but acquired antibodies to measles or rubella antibodies ($\boldsymbol{c}_{\boldsymbol{a,m}}$ and $\boldsymbol{c}_{\boldsymbol{a,r}}$), the vaccination coverage ($\boldsymbol{v}_{\boldsymbol{a}}$) and the immunogenicity for the measles and rubella components of the vaccine ($\boldsymbol{e}_{\boldsymbol{m}}$and $\boldsymbol{e}_{\boldsymbol{r}}\boldsymbol{)}$used to generate the data. The average values for $\boldsymbol{e}_{\boldsymbol{m}}$and $\boldsymbol{e}_{\boldsymbol{r}}$ were 75% and 90% respectively. Columns 7-11 show the numbers of people who were positive and/or negative for measles and/or rubella antibodies. Proportions are provided to 4 decimal places. $\boldsymbol{c}_{\boldsymbol{a,m}}$ and $\boldsymbol{c}_{\boldsymbol{a,r}}$ were calculated as $\boldsymbol{c}_{\boldsymbol{a,m=}}\boldsymbol{c}_{\boldsymbol{a-1,m}}\boldsymbol{+R}$, where R is a random number, sampled from the normal distribution with a mean of zero and standard deviation of 0.1. $\boldsymbol{c}_{\boldsymbol{a,r}}$ was calculated similarly.

a) Simulated data – low average vaccine coverage (77%)

| **Age (years)** | $\boldsymbol{c}_{\boldsymbol{a,m}}$ | $\boldsymbol{c}_{\boldsymbol{a,r}}$ | $\boldsymbol{v}_{\boldsymbol{a}}$ | $\boldsymbol{e}_{\boldsymbol{m}}$ | $\boldsymbol{e}_{\boldsymbol{r}}$ | $\boldsymbol{n}_{\boldsymbol{a++}}$ | $\boldsymbol{n}_{\boldsymbol{a+-}}$ | $\boldsymbol{n}_{\boldsymbol{a-+}}$ | $\boldsymbol{n}_{\boldsymbol{a--}}$ | $\boldsymbol{N}_{\boldsymbol{a}}$ |
| --- | --- | --- | --- | --- | --- | --- | --- | --- | --- | --- |
| 5 | 0.45 | 0.1836 | 0.7488 | 0.7463 | 0.9151 | 17 | 4 | 3 | 3 | 27 |
| 6 | 0.4784 | 0.3637 | 0.777 | 0.7511 | 0.8718 | 22 | 4 | 4 | 3 | 33 |
| 7 | 0.432 | 0.408 | 0.7624 | 0.7731 | 0.8935 | 25 | 4 | 5 | 3 | 37 |
| 8 | 0.652 | 0.5422 | 0.7625 | 0.7492 | 0.9489 | 25 | 3 | 4 | 1 | 33 |
| 9 | 0.7353 | 0.4965 | 0.7758 | 0.7375 | 0.8802 | 17 | 3 | 2 | 1 | 23 |
| 10 | 0.6139 | 0.4911 | 0.7649 | 0.7621 | 0.8978 | 28 | 4 | 4 | 2 | 38 |
| 11 | 0.7612 | 0.5566 | 0.7694 | 0.7466 | 0.9576 | 24 | 3 | 2 | 1 | 30 |
| 12 | 0.6828 | 0.5962 | 0.7672 | 0.7629 | 0.9309 | 24 | 3 | 3 | 1 | 31 |
| 13 | 0.6464 | 0.6666 | 0.7828 | 0.761 | 0.9073 | 23 | 2 | 3 | 1 | 29 |
| 14 | 0.7334 | 0.697 | 0.7675 | 0.7561 | 0.9023 | 33 | 3 | 4 | 1 | 41 |
| 15 | 0.7633 | 0.6982 | 0.7618 | 0.746 | 0.9219 | 56 | 5 | 6 | 1 | 68 |
| 16 | 0.8138 | 0.7859 | 0.7625 | 0.7083 | 0.8849 | 65 | 4 | 6 | 1 | 76 |
| 17 | 0.7813 | 0.7034 | 0.7664 | 0.7406 | 0.8729 | 61 | 6 | 6 | 1 | 74 |
| 18 | 0.6387 | 0.7551 | 0.766 | 0.7478 | 0.9004 | 46 | 3 | 8 | 1 | 58 |
| 19 | 0.6346 | 0.7565 | 0.7768 | 0.7158 | 0.8844 | 38 | 3 | 7 | 1 | 49 |
| 20 | 0.7139 | 0.7669 | 0.7658 | 0.7304 | 0.8928 | 17 | 1 | 2 | 0 | 20 |
| 21 | 0.5941 | 0.8058 | 0.7737 | 0.7696 | 0.8946 | 14 | 1 | 2 | 0 | 17 |

Table A.1 continued

b) Simulated data assuming medium average vaccine coverage (85%)

| **Age (years)** | $\boldsymbol{c}_{\boldsymbol{a,m}}$ | $\boldsymbol{c}_{\boldsymbol{a,r}}$ | $\boldsymbol{v}_{\boldsymbol{a}}$ | $\boldsymbol{e}_{\boldsymbol{m}}$ | $\boldsymbol{e}_{\boldsymbol{r}}$ | $\boldsymbol{n}_{\boldsymbol{a++}}$ | $\boldsymbol{n}_{\boldsymbol{a+-}}$ | $\boldsymbol{n}_{\boldsymbol{a-+}}$ | $\boldsymbol{n}_{\boldsymbol{a--}}$ | $\boldsymbol{N}_{\boldsymbol{a}}$ |
| --- | --- | --- | --- | --- | --- | --- | --- | --- | --- | --- |
| 5 | 0.45 | 0.3791 | 0.8444 | 0.7866 | 0.9021 | 20 | 2 | 3 | 2 | 27 |
| 6 | 0.5472 | 0.4009 | 0.854 | 0.7504 | 0.8688 | 24 | 4 | 4 | 2 | 34 |
| 7 | 0.6393 | 0.4227 | 0.8356 | 0.7735 | 0.8903 | 28 | 4 | 3 | 1 | 36 |
| 8 | 0.6579 | 0.5259 | 0.8516 | 0.7526 | 0.9355 | 27 | 2 | 3 | 1 | 33 |
| 9 | 0.6359 | 0.4995 | 0.8456 | 0.7506 | 0.8928 | 18 | 2 | 2 | 1 | 23 |
| 10 | 0.6872 | 0.6599 | 0.8535 | 0.7526 | 0.9105 | 32 | 2 | 4 | 1 | 39 |
| 11 | 0.844 | 0.6522 | 0.8406 | 0.7326 | 0.897 | 26 | 2 | 2 | 0 | 30 |
| 12 | 0.8979 | 0.6832 | 0.8475 | 0.763 | 0.8592 | 27 | 2 | 1 | 0 | 30 |
| 13 | 0.8325 | 0.6582 | 0.8685 | 0.6972 | 0.9148 | 25 | 2 | 2 | 0 | 29 |
| 14 | 0.7144 | 0.6134 | 0.8687 | 0.7295 | 0.8811 | 34 | 3 | 4 | 1 | 42 |
| 15 | 0.6749 | 0.6221 | 0.8351 | 0.7887 | 0.9006 | 56 | 5 | 6 | 2 | 69 |
| 16 | 0.558 | 0.8206 | 0.8421 | 0.7415 | 0.8939 | 61 | 2 | 12 | 1 | 76 |
| 17 | 0.5389 | 0.6425 | 0.8341 | 0.7519 | 0.8859 | 57 | 5 | 10 | 2 | 74 |
| 18 | 0.6372 | 0.8158 | 0.8572 | 0.7795 | 0.9736 | 50 | 1 | 6 | 1 | 58 |
| 19 | 0.5884 | 0.7218 | 0.8496 | 0.7595 | 0.9053 | 40 | 2 | 6 | 1 | 49 |
| 20 | 0.3892 | 0.7799 | 0.8372 | 0.7419 | 0.9029 | 16 | 1 | 4 | 1 | 22 |
| 21 | 0.4156 | 0.7821 | 0.8312 | 0.7329 | 0.9111 | 13 | 0 | 3 | 0 | 16 |

Table A.1 continued

c) Simulated data, assuming high average vaccine coverage (97%)

| **Age (years)** | $\boldsymbol{c}_{\boldsymbol{a,m}}$ | $\boldsymbol{c}_{\boldsymbol{a,r}}$ | $\boldsymbol{v}_{\boldsymbol{a}}$ | $\boldsymbol{e}_{\boldsymbol{m}}$ | $\boldsymbol{e}_{\boldsymbol{r}}$ | $\boldsymbol{n}_{\boldsymbol{a++}}$ | $\boldsymbol{n}_{\boldsymbol{a+-}}$ | $\boldsymbol{n}_{\boldsymbol{a-+}}$ | $\boldsymbol{n}_{\boldsymbol{a--}}$ | $\boldsymbol{N}_{\boldsymbol{a}}$ |
| --- | --- | --- | --- | --- | --- | --- | --- | --- | --- | --- |
| 5 | 0.45 | 0.4141 | 0.9868 | 0.7734 | 0.8986 | 22 | 1 | 3 | 0 | 26 |
| 6 | 0.6908 | 0.4188 | 0.9713 | 0.7246 | 0.9103 | 28 | 2 | 3 | 0 | 33 |
| 7 | 0.6539 | 0.4913 | 0.9787 | 0.7654 | 0.9167 | 32 | 2 | 3 | 0 | 37 |
| 8 | 0.6131 | 0.5175 | 0.9616 | 0.7369 | 0.8838 | 27 | 2 | 3 | 0 | 32 |
| 9 | 0.5844 | 0.4812 | 0.9681 | 0.734 | 0.9327 | 19 | 1 | 3 | 0 | 23 |
| 10 | 0.5492 | 0.5993 | 0.9604 | 0.7572 | 0.8721 | 32 | 2 | 4 | 0 | 38 |
| 11 | 0.5117 | 0.5149 | 0.9739 | 0.7574 | 0.8925 | 25 | 2 | 3 | 0 | 30 |
| 12 | 0.6703 | 0.679 | 0.976 | 0.7536 | 0.9325 | 28 | 1 | 3 | 0 | 32 |
| 13 | 0.7809 | 0.6188 | 0.9601 | 0.7495 | 0.8973 | 26 | 1 | 2 | 0 | 29 |
| 14 | 0.7959 | 0.5531 | 0.9755 | 0.73 | 0.9142 | 37 | 2 | 2 | 0 | 41 |
| 15 | 0.9052 | 0.6764 | 0.9692 | 0.7515 | 0.8985 | 64 | 3 | 2 | 0 | 69 |
| 16 | 0.9023 | 0.6953 | 0.9815 | 0.7297 | 0.8994 | 71 | 3 | 2 | 0 | 76 |
| 17 | 0.8645 | 0.6827 | 0.9806 | 0.763 | 0.9142 | 69 | 2 | 2 | 0 | 73 |
| 18 | 0.694 | 0.8211 | 0.9714 | 0.7307 | 0.8947 | 52 | 1 | 5 | 0 | 58 |
| 19 | 0.8357 | 0.7267 | 0.9667 | 0.7499 | 0.8553 | 45 | 2 | 2 | 0 | 49 |
| 20 | 0.886 | 0.7512 | 0.9554 | 0.7399 | 0.9237 | 20 | 1 | 1 | 0 | 22 |
| 21 | 0.9481 | 0.8621 | 0.9716 | 0.7282 | 0.8605 | 16 | 0 | 0 | 0 | 16 |

Figure A.1: Comparison between base-case estimates (>95% vaccination coverage) and best-fitting estimates (>80% vaccination coverage) obtained by maximum likelihood fitting with simplified assumptions A. Age-specific proportion of unvaccinated people who had acquired detectable antibodies to measles (*c_a,m_*) or rubella (*c_a,r_*) through natural infection (measles and rubella) or vaccination (measles only) . B. Comparison between the observed data (crosses, with bars for the 95% CI) and the % estimated to be positive or negative for measles and/or rubella antibodies. The solid squares and shaded areas respectively show the point estimate and 95% credible range respectively of the calculated estimates.

Figure A.2:. Findings from applying Bayesian Melding to simulated data for 5-14 year olds, assuming an average vaccine coverage of 77%. A. Age-specific proportion of unvaccinated people who had acquired detectable antibodies to measles (*c_a,m_*) or rubella (*c_a,r_*) through natural infection (measles and rubella) or vaccination (measles only) . B. Comparison between the simulated data (crosses, with bars for the 95% CI) and the % estimated to be positive or negative for measles and/or rubella antibodies. The solid squares and shaded areas respectively show the point estimate and 95% credible range respectively of the calculated estimates.

Figure A.3:. Findings from applying Bayesian Melding to simulated data for 5-14 year olds, assuming an average vaccine coverage of 85%. A. Age-specific proportion of unvaccinated people who had acquired detectable antibodies to measles (*c_a,m_*) or rubella (*c_a,r_*) through natural infection (measles and rubella) or vaccination (measles only) . B. Comparison between the simulated data (crosses, with bars for the 95% CI) and the % estimated to be positive or negative for measles and/or rubella antibodies. The solid squares and shaded areas respectively show the point estimate and 95% credible range respectively of the calculated estimates.

Figure A.4:. Findings from applying Bayesian Melding to simulated data for 5-14 year olds, assuming an average vaccine coverage of 97%. A. Age-specific proportion of unvaccinated people who had acquired detectable antibodies to measles (*c_a,m_*) or rubella (*c_a,r_*) through natural infection (measles and rubella) or vaccination (measles only) . B. Comparison between the simulated data (crosses, with bars for the 95% CI) and the % estimated to be positive or negative for measles and/or rubella antibodies. The solid squares and shaded areas respectively show the point estimate and 95% credible range respectively of the calculated estimates.

**References**

1 Gay, N. J. *A method for estimating coverage of a multivalent vaccine from antibody prevalence data: application to MMR vaccine in 3 European countries* (unpublished).

2 Altmann, D. & Altmann, K. Estimating vaccine coverage by using computer algebra. *IMA J Math Appl Med Biol* **17**, 137-146 (2000).

3 Goeyvaerts, N. *et al.* Estimating vaccination coverage for the trivalent measles-mumps-rubella vaccine from trivariate serological data. *Stat Med* **31**, 1432-1449 (2012).

4 Shkedy, Z., Aerts, M., Molenberghs, G., Beutels, P. & Van Damme, P. Modelling age-dependent force of infection from prevalence data using fractional polynomials. *Stat Med* **25**, 1577-1591, (2006).
